# Delayed feedback control for chaotic vibration in nonlinear impact dynamics of bouncing agricultural tractor

**DOI:** 10.1038/s41598-023-37916-1

**Published:** 2023-07-02

**Authors:** Masahisa Watanabe, Kenshi Sakai

**Affiliations:** Division of Environmental and Agricultural Engineering, Institute of Agriculture, Tokyo University of Agriculture and Technology, 3-5-8 Saiwai-cho, Fuchu, Tokyo 183-8509 Japan

**Keywords:** Mechanical engineering, Engineering

## Abstract

Agricultural tractors often lose contact and recollide with the ground surface while driving on narrow paddy fields and bumpy farm roads owing to excessive vibrations. These nonlinear impact dynamics can cause chaotic vibrations during tractor operation. Chaotic vibrations are random complex motions that can deteriorate tractor stability and lead to tractor overturning accidents, causing damage to machinery and risk of injury to the operator. This study investigates the theoretical feasibility of chaos control to eliminate chaotic vibrations in tractor dynamics. Delayed feedback (DF) control is employed to eliminate complex vibrations in tractor dynamics. First, the frequency response, bifurcation diagram, and largest Lyapunov exponent are obtained to investigate the nonlinear dynamics of the tractor and identify the parametric region in which chaotic vibrations occur. Subsequently, the DF control is designed based on the trial-and-error method and applied to the tractor dynamics as the driving force control input. The numerical results demonstrate that the DF control can successfully eliminate chaotic vibration and reduce the vibration level. Therefore, this study is expected to contribute to improving the tractor safety by reducing the risk of overturning.

## Introduction

An agricultural tractor is a self-propelled vehicle used for various farm operations, such as tillage, transportation, seeding, and towing. As tractors are mainly operated off-road, on farm fields and unpaved roads, they suffer from severe vibrations excited by undulating terrains. As the working speed of the tractor increases, vibrations become more problematic. Owing to severe vibrations, the operator may lose control of the tractor, and an overturning accident may occur. Tractor overturning is a major cause of farm fatalities worldwide^1^. In Japan, 53 out of 270 fatal farm accidents in 2020 were caused by tractor overturning^2^. Tractor dynamics and control have been investigated to predict vibrations and prevent overturning^3–11^. Therefore, active tractor control, including active steering control^12,13^, active driving force control^14^, and sliding mode control^15^, is intensively studied.

In Japan, small agricultural tractors are generally used on narrow paddy fields and bumpy farm roads. Therefore, the vibration problem is more serious than that of a large tractor on relatively flat ground. In a small tractor, excessive and violent vibrations often cause the tractor tires to lose contact and recollide with the ground. These impact dynamics are similar to bouncing ball dynamics and are a strong nonlinear element in tractor dynamics^16–19^. Nonlinear tractor dynamics often lead to chaotic vibrations^20–26^. Chaotic vibrations or chaos are random-like motions, even though the motions are generated by deterministic dynamics such as motion equations. It is difficult to predict chaotic vibrations because of their inherent property of sensitive dependence on initial conditions and parameters. Thus, chaotic vibrations deteriorate tractor stability and lead to tractor overturning, causing damage to machinery and risk of injury to operators. In addition, chaotic vibrations cause undesirable peaks in the spectrum such as subharmonics, which are generally not considered in linear vehicle design theory. As well as agricultural tractor, chaotic vibration sometimes appears in automotive dynamics. Several studies on chaotic vibration in automotive dynamics have been conducted for safety and performance improvement^27–31^. Therefore, chaotic vibration is a significant research topic in both tractor and automotive engineering.

Chaos is a generally undesirable phenomenon observed in various systems, such as electronic and mechanical systems, and should be eliminated via the control input. Thus far, many studies have been conducted on chaos control methods to eliminate chaos in nonlinear dynamics. Ott, Grebogi, and York^32^ developed a chaos control method that can eliminate chaos in dynamics by making a small time-dependent perturbation (OGY control). OGY control is a time-discrete control method that requires a permanent computer analysis of the state of the system. However, because of the abovementioned properties, OGY control can lead to an occasional burst of the system when a large noise occurs in the system. Pyragas^33,34^ developed the delayed feedback control (DF control), a time-continuous control method, to eliminate chaos. The DF control method involves reference-free control that is calculated based on the difference between the current state of the system and the time-delayed state. As DF control is easy to implement, it is applied to various experimental and theoretical systems such as electronic oscillators^35^, mechanical pendulums^36^, helicopter rotor blades^37^, and car-following traffic^38^. Even though these practical applications suggest that DF control has a potential to apply to tractor dynamics, DF control for agricultural tractor has not been intensively investigated yet.

The objective of this study is to investigate the theoretical feasibility of DF control in eliminating chaotic vibrations in tractor impact dynamics. First, a nonlinear tractor dynamic model with three degrees of freedom is considered. Subsequently, the DF control for a tractor is introduced as an active driving force control. The frequency response, bifurcation diagram, and largest Lyapunov exponent are obtained to identify the parametric region in which chaotic vibrations occur. DF control is applied to the chaotic vibration in the tractor. The remainder of this paper is organized as follows. In “Nonlinear impact dynamic model for agricultural tractor” and “Delayed feedback control design” sections introduce the nonlinear tractor dynamic model and the DF control for the tractor, respectively. The numerical results are described in “Numerical results” section. Finally, in “Conclusion” section presents the conclusions. The innovative point of the present study is to demonstrate numerical feasibility of DF control application to agricultural tractor and to provide basic policy for tuning DF control parameters specific to agricultural tractor applications.

## Nonlinear impact dynamic model for agricultural tractor

**Figure 1:**
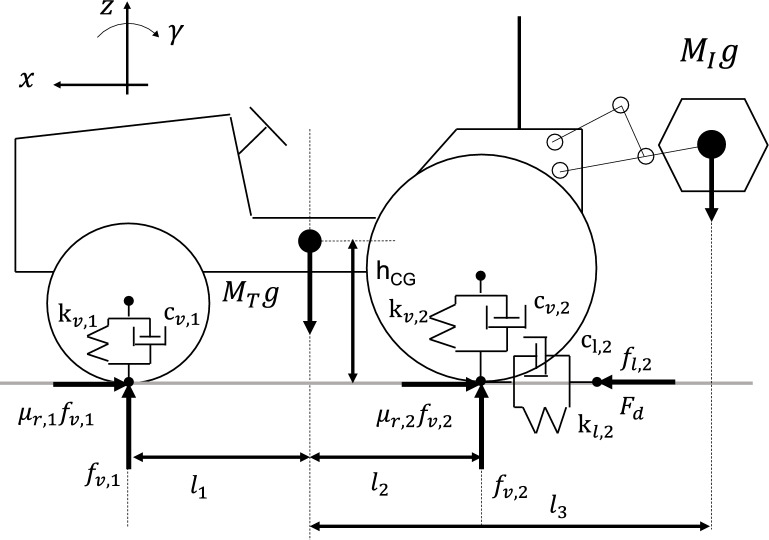
Schematic of the tractor dynamic model with three degrees of freedom; vertical motion *z*, longitudinal motion *x*; pitch motion *γ.*

This section explains the nonlinear impact dynamic model of the agricultural tractors used in this study. Figure 1 shows a schematic of the tractor model with three degrees of freedom.

The tractor model has three degrees of freedom: vertical motion *z*, longitudinal motion *x*, and pitch motion *γ*. According to the force balance method, the motion equations of the developed model are derived according to Eqs. (1)–(3), as follows:

1 $$\left( {M_{T} + M_{I} } \right)\ddot{z} = f_{v,1} + f_{v,2} - \left( {M_{T} + M_{I} } \right)g,$$

2 $$(I_{yy} + M_{I} l_{3}^{2} )\ddot{\gamma } = l_{1} f_{v,1} - l_{2} f_{v,2} + \left( {F_{d} - \mu_{r,1} f_{v,1} - \mu_{r,2} f_{v,2} + f_{l,2} } \right)h_{CG} ,$$

3 $$\left( {M_{T} + M_{I} } \right)\ddot{x} = F_{d} - \mu_{r,1} f_{v,1} - \mu_{r,2} f_{v,2} + f_{l,2} ,$$

where M_T_ is the mass of the tractor body; M_I_ is the mass of the implement; g is the gravitational acceleration; h_CG_ is the height of the center of gravity; *f*_v,1_ and *f*_v,2_ are the vertical forces acting on the front and rear tires, respectively; *f*_l,2_ is the longitudinal force acting on the rear tire; *F*_d_ is the driving force on the rear tire. In this study, a rear wheel drive tractor was assumed, and thus, no driving force was exerted on the front wheel.

In vertical and longitudinal tire force modeling, tires are expressed by the Kelvin–Voigt model (linear spring and damper) when the tire makes contact with the ground. The tire forces become zero when the tire loses contact with the ground. These impact nonlinearities are modeled using switching equations, involving bouncing ball dynamics. The vertical and longitudinal forces are calculated using Eqs. (4)–(6), as follows:

4 $$f_{v,1} = \left\{ {\begin{array}{*{20}l} { - {\text{k}}_{{{\text{v}},1}} \left( {z_{1} - d_{1} } \right) - {\text{c}}_{{{\text{v}},1}} \left( {\dot{z}_{1} - \dot{d}_{1} } \right), \;\;\;\; {\text{if}}\;{\text{ the }}\;{\text{front }}\;{\text{wheel }}\;{\text{contacts }}\;{\text{the }}\;{\text{ground}}} \hfill \\ {0, \;\;\;\;{\text{if }}\;{\text{the }}\;{\text{wheel }}\;{\text{loses }}\;{\text{contact }}\;{\text{with }}\;{\text{the }}\;{\text{ground}}} \hfill \\ \end{array} } \right.,$$

5 $$f_{v,2} = \left\{ {\begin{array}{*{20}l} { - {\text{k}}_{{{\text{v}},2}} \left( {z_{2} - d_{2} } \right) - {\text{c}}_{{{\text{v}},2}} \left( {\dot{z}_{2} - \dot{d}_{2} } \right),\;\;\;\;{\text{if}}\;{\text{ the}}\;{\text{ rear}}\;{\text{ wheel}}\;{\text{ contacts}}\;{\text{ the}}\;{\text{ ground}}} \hfill \\ {0, \;\;\;\;{\text{if}}\;{\text{ the}}\;{\text{ rear}}\;{\text{ wheel}}\;{\text{ loses }}\;{\text{contact}}\;{\text{ with}}\;{\text{ the}}\;{\text{ ground}}} \hfill \\ \end{array} } \right.,$$

6 $$f_{l,2} = \left\{ {\begin{array}{*{20}l} { - {\text{k}}_{{{\text{l}},2}} x - {\text{c}}_{{{\text{l}},2}} \dot{x},\;\;\;\; {\text{if}}\;{\text{ the}}\;{\text{ rear}}\;{\text{ wheel}}\;{\text{ contacts }}\;{\text{the }}\;{\text{ground}}} \hfill \\ {0,\;\;\;\; {\text{if }}\;{\text{the}}\;{\text{ rear}}\;{\text{ wheel }}\;{\text{loses }}\;{\text{contact }}\;{\text{with }}\;{\text{the }}\;{\text{ground}}} \hfill \\ \end{array} } \right.,$$

where *k*_v,1_, *k*_v,2_, and *k*_l, 2_ are the stiffnesses of the front vertical, rear vertical, and rear longitudinal wheels, respectively; *c*_*v,*1_, *c*_*v.*2_, and *c*_l, 2_ are the damping coefficients of the front vertical, rear vertical, and rear longitudinal wheels, respectively; *z*_1_ and *z*_2_ are the vertical displacements of the front and rear wheels, respectively; *x* is the longitudinal displacement of the rear wheel; *d*_1_ and *d*_2_ denote the front and rear road elevation, respectively. The bouncing process of an agricultural tractor was explained by Watanabe and Sakai^23^. *z*_1_ and *z*_2_ are calculated using Eqs. (7) and (8), respectively:

7 $$z_{1} = z + \left( {l_{1} + l_{3} \frac{{M_{I} }}{{M_{T} + M_{I} }}} \right)\gamma ,$$

8 $$z_{2} = z - (l_{2} - l_{3} \frac{{M_{I} }}{{M_{T} + M_{I} }})\gamma .$$

In this study, the road excitation functions are defined as sine functions, as expressed by Eqs. (9) and (10):

9 $$d_{1} = d_{0} \sin \left( {2\pi f_{t} t} \right),$$

10 $$d_{2} = d_{0} \sin \left( {2\pi f_{t} \left( {t - \frac{{l_{1} + l_{2} }}{V}} \right)} \right),$$

where *d*_0_, V, and *f*_t_ is the road amplitude, the travel velocity of the tractor, the forcing frequency respectively. The road amplitude *d*_0_ was set as 0.015 m in the numerical simulations.

## Delayed feedback control design

**Figure 2:**
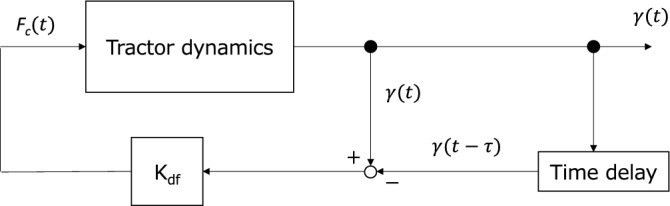
Block diagram of delayed feedback control based on Pyragas^33^.

This section explains the DF control of driving forces. The driving forces on the rear wheel are calculated from Eq. (11), as follows:

11 $$F_{{\text{d}}} = \left\{ {\begin{array}{*{20}l} {\mu_{r,1} f_{v,1} + \mu_{r,2} f_{v,2} + F_{c} ,\;\;\;\;{\text{if}}\;{\text{the}}\;{\text{rear}}\;{\text{ wheel }}\;{\text{contacts}}\;{\text{ the }}\;{\text{ground}}} \hfill \\ {0,\;\;\;\;{\text{if}}\;{\text{ the }}\;{\text{rear }}\;{\text{wheel}}\;{\text{loses}}\;{\text{contact}}\;{\text{with}}\;{\text{the}}\;{\text{ground}}} \hfill \\ \end{array} } \right.,$$

where the term μ_r,1_* f*_v,1_ + μ_r,1_* f*_v,1_ denotes the force necessary for overcoming motion resistance, and *F*_c_ is a DF control force term. The driving force *F*_d_ should be zero when the wheel loses contact with the ground. The DF control force *F*_c_ is calculated based on the method described by Pyragas^33^. DF control can stabilize chaotic vibrations by converting them into periodic vibrations. Figure 2 shows a block diagram of the DF control implemented in tractor dynamics.

In DF control, the feedback term is calculated based on the signal difference $\gamma \left( t \right) - \gamma \left( {t - \tau } \right)$ between the current state of the system and the state of the system delayed τ. As the pitch angle γ(t) can be easily measured in a practical situation, it is used as the representative signal of tractor dynamics in this study. The delayed feedback control forces are proportional to the signal difference, as shown in Eq. (12):

12 $$F_{{\text{c}}} = {\text{K}}_{{{\text{df}}}} \left( {\gamma \left( t \right) - \gamma \left( {t - \tau } \right)} \right),$$

where K_df_ is the DF gain, and τ is the time delay for DF control. In DF control, unstable periodic orbits embedded in a strange attractor are stabilized by input perturbations A general determination methodology for the DF parameters has not been established thus far. The time delay τ and gain K_df_ are tuned based on try-and-error. Table 1 shows the parameter specifications of the small farm tractor used in the present study.

**Table 1:** Nomenclature and parameter specifications of the small tractor for the numerical experiments^39^.

Parameters	Symbol	Value	Unit
Vertical stiffness of front wheel	*k*_1, v_	304	kN m^-1^
Vertical stiffness of rear wheel	*k*_2, v_	372	kN m^-1^
Longitudinal stiffness of rear wheel	*k*_2, l_	316	kN m^-1^
Vertical damping coefficient of front wheel	*c*_1, v_	5500	N s m^-1^
Vertical damping coefficient of rear wheel	*c*_2, v_	6690	N s m^-1^
Longitudinal damping coefficient of rear wheel	*c*_2, l_	6400	N s m^-1^
Mass of tractor body	*M*_T_	874	kg
Mass of implement	*M*_I_	149	kg
Pitch moment of inertia	*I*_yy_	320	kg m^-2^
Distance between the center of gravity of the tractor body and the front wheel	*l*_1_	0.78	m
Distance between the center of gravity of the tractor body and rear wheel	*l*_2_	0.587	m
Distance between the center of gravity of the tractor body and the center of gravity of the implement	*l*_3_	1.549	m
The wheelbase of the tractor	*WB*	1.35	m
The height of the center of gravity from the ground	h_CG_	0.586	m
Motion resistance coefficient of front wheel	μ_r,1_	0.12	–
Motion resistance coefficient of rear wheel	μ_r,2_	0.12	–
Sinus road function amplitude	d_0_	0.015	m
Vertical motion	*z*	–	m
Vertical velocity	*v*_z_	–	m s^-1^
Pitch motion	*γ*	–	rad
Longitudinal motion	*x*	–	m
Vertical force on front wheel	*f*_*v*_,_1_	–	kN
Vertical force on rear wheel	*f*_v,2_	–	kN
Longitudinal force on rear wheel	*f*_l,2_	–	kN
Driving force	*F*_d_	–	kN
Delayed feedback control force	*F*_c_	–	kN
Vertical motion of front wheel	*z*_1_	–	m
Vertical motion of rear wheel	*z*_2_	–	m
Gravitational acceleration	g	–	g
Travel velocity	V	–	m s^-1^
Largest Lyapunov exponent	λ_1_	–	–
Forcing frequency	*f*_t_	–	Hz
Primary frequency in the spectrum	*f*_p_	–	Hz
Time delay for DF control	*τ*	–	s
Gain for DF control	K_df_	–	kN rad^-1^

## Numerical results

### Chaotic vibrations in tractor dynamics

In this section, the observations on chaotic vibration in tractor dynamics are presented. In this study, delayed feedback control is applied to eliminate chaotic vibration. First, the frequency response, bifurcation diagram, and largest Lyapunov exponent λ_1_ are obtained to identify the parametric region in which chaotic vibration occurs. Frequency response is typically used to analyze the response of a dynamical system. In this study, the forcing frequency *f*_t_ is varied from 1.0 to 7.0 Hz, and the maximum value of vertical displacement *z* is plotted at each forcing frequency.

In nonlinear dynamics, bifurcation is a structural change that describes the stability of a periodic orbit^40^. The bifurcation diagram is widely used to visualize orbit stability and easily identify the parametric region in which chaotic vibration occurs. In this study, a bifurcation diagram is obtained by plotting the Poincaré point of the vertical displacement *z* concerning the forcing frequency *f*_t_. In the bifurcation diagram, a single bifurcation plot indicates one periodic vibration, whereas multiple complex plots indicate multiple complex vibrations.

The largest Lyapunov exponent λ_1_ quantifies the divergence or convergence rate of the trajectory in the phase space and is generally used to identify chaos in dynamics^41^. The negative, zero, and positive largest Lyapunov exponents indicate a fixed point (no vibration), normal periodic vibration, and chaotic vibration, respectively. In this study, the largest Lyapunov exponent λ_1_ is calculated based on vertical displacement time series data^42,43^. Based on the time delay embedding technique, the attractor is reconstructed, and the Jacobian G(t) is estimated at each time point t. Given a set of unit vectors that are orthogonal to each other, denoted as u_i_(t) (where i = 1, 2, …, m), each unit vector is transformed to e_i_(t + 1) using the estimated Jacobian G(t). Here, m represents the embedding dimension. The calculation formula for the transformation is as follows:

13 $${\text{e}}_{{\text{i}}} \left( {t + 1} \right) = G\left( t \right)u_{i} \left( t \right),$$

In each iteration, Eq. (13) is orthonormalized by Gram-Schmit method.

14 $${\text{e}}_{{\text{i}}}^{^{\prime}} \left( {t + 1} \right) = e_{i} \left( {t + 1} \right) - \mathop \sum \limits_{j = 1}^{i - 1} \left[ {e_{i} \left( {t + 1} \right) \cdot u_{j} \left( {t + 1} \right)} \right]u_{j} \left( {t + 1} \right)$$

15 $${\text{u}}_{{\text{i}}} \left( {t + 1} \right) = \frac{{e_{i}^{^{\prime}} \left( {t + 1} \right)}}{{\left| {e_{i}^{^{\prime}} \left( {t + 1} \right)} \right|}}$$

Lyapunov exponent is calculated based on e’_i_(t) as follows:

16 $${\uplambda }_{{\text{i}}} = \mathop {\lim }\limits_{N \to \infty } \frac{{\mathop \sum \nolimits_{t = 0}^{N - 1} {\text{ln}}\left( {e_{i}^{^{\prime}} \left( t \right)} \right)}}{N},$$

where N is data size of the time series.

**Figure 3:**
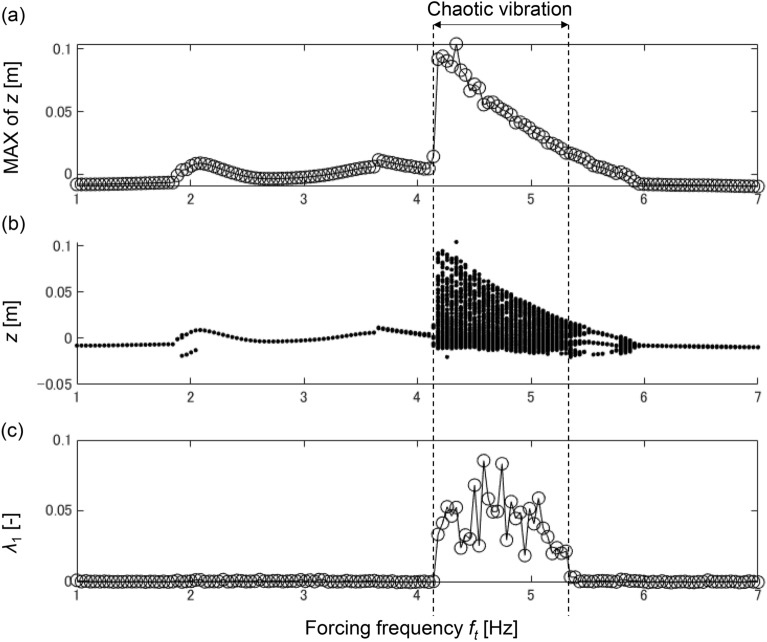
(**a**) Frequency response, (**b**) Bifurcation diagram, (**c**) Largest Lyapunov Exponent λ_1_.

Figure 3a–c show the frequency response, bifurcation diagram, and largest Lyapunov exponent λ_1_, respectively.

Figure 3 shows that the frequency response is continuous, and the bifurcation diagram indicates periodic vibration from 1.0 to 4.1 Hz. In addition, the largest Lyapunov exponent λ_1_ is zero in the forcing frequency range of 1.0–4.1 Hz, indicating that periodic vibration occurs from 1.0 to 4.1 Hz. At the point of 4.1 Hz, the frequency response increases discontinuously. This discontinuous response is a typical feature of nonlinear dynamics. The bifurcation plot shows multiple complex plots, and the largest Lyapunov exponent λ_1_ is positive from 4.1 to 5.4 Hz, indicating that chaotic vibration occurs in the range of 4.1–5.4 Hz. In the frequency region above 5.4 Hz, the largest Lyapunov exponent λ_1_ is zero, indicating that periodic vibrations occur in this frequency band. The bifurcation plot becomes complex from 5.8 to 6.0 Hz with λ_1_ = 0, indicating that quasi-periodic vibration occurs from 5.8 to 6.0 Hz.

**Figure 4:**
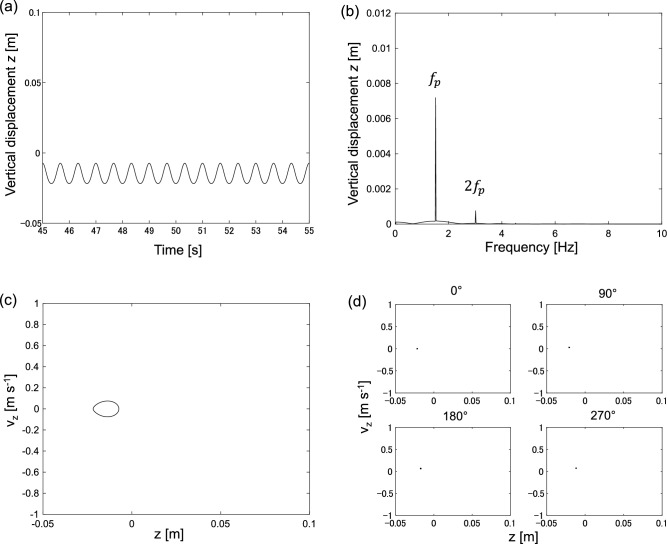
Periodic vibration at 1.5 Hz. (**a**) Time-series of vertical displacement *z*, (**b**) Fourier spectrum of vertical displacement *z*, (**c**) Phase plane spanned by vertical displacement *z* and vertical velocity *v*_z_, (**d**) Poincaré section spanned by vertical displacement *z* and vertical velocity *v*_z_.

Typical examples of periodic, quasiperiodic, and chaotic vibrations are presented as follows. Figure 4 shows a typical example of periodic vibration at 1.5 Hz.

Figure 4a shows the time series of vertical displacement is periodic. Figure 4b shows the Fourier spectrum of the vertical displacement *z*. In the spectrum, the primary frequency *f*_p_ is equal to a forcing frequency of 1.5 Hz. Figure 4c shows the motion trajectory in the phase plane spanned by vertical displacement and vertical velocity. The trajectory is a limit cycle corresponding to periodic motion. Figure 4d shows the Poincaré section of the trajectory in the phase plane. The Poincaré section is typically employed to study the trajectory structure in the phase plane; it can be derived via stroboscopic monitoring of the point in the phase plane. Therefore, one point in the Poincaré section corresponds to one specific period. In Fig. 4d, the Poincaré section is derived at different angles, that is, 0°, 90°, 180°, and 270°. All the Poincaré sections show a single point in the phase plane. This implies that the vibration is periodic.

**Figure 5:**
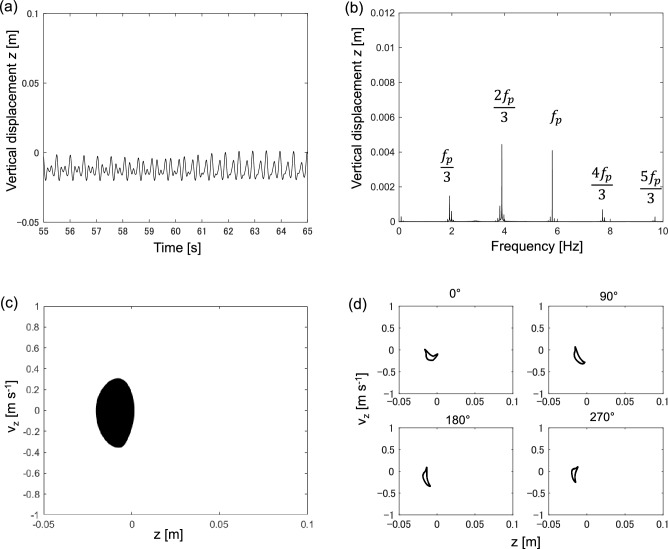
Quasi-periodic vibration at 5.8 Hz. (**a**) Time-series of vertical displacement *z*, (**b**) Fourier spectrum of vertical displacement *z*, (**c**) Phase plane spanned by vertical displacement *z* and vertical velocity *v*_z_, (**d**) Poincaré section spanned by vertical displacement *z* and vertical velocity *v*_z_.

Figure 5 shows a typical example of quasi-periodic vibration at 5.8 Hz.

In Fig. 5a, the time series of vertical displacement demonstrates multiple periodic vibrations. Multiple peaks are observed in the Fourier spectrum, corresponding to the primary frequency *f*_p_ = 5.8 Hz, subharmonics *f*_p_/3 = 1.93 Hz, and ultra-subharmonics of 2*f*_p_/3 = 3.86 Hz, 4*f*_p_/3 = 7.72 Hz, and 5*f*_p_/3 = 9.65 Hz. In Fig. 5c, the trajectory is a torus in the phase plane. A closed loop is observed in the Poincaré section in Fig. 5d. This serves as evidence for the existence of quasi-periodic vibration.

**Figure 6:**
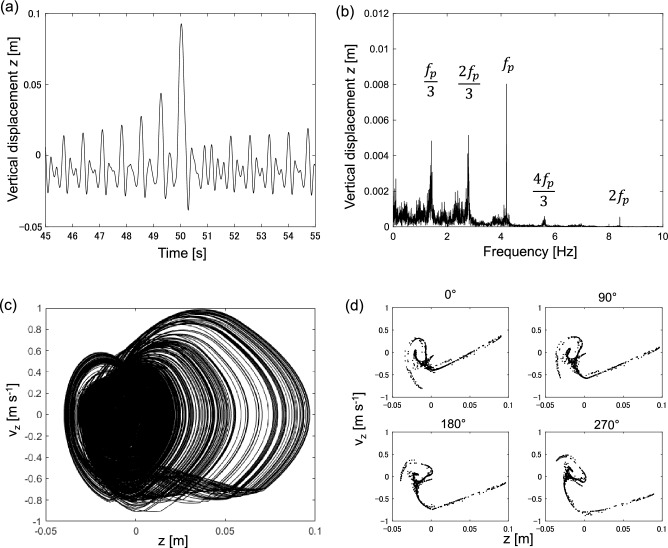
Chaotic vibration at 4.2 Hz. (**a**) Time series of vertical displacement *z*, (**b**) Fourier spectrum of vertical displacement *z*, (**c**) Phase plane spanned by vertical displacement *z* and vertical velocity *v*_z_, (**d**) Poincaré section spanned by vertical displacement *z* and vertical velocity *v*_z_.

Figure 6a shows that the vertical displacement vibration is complex and random. The noise level is significantly high in the Fourier spectrum shown in Fig. 6b, even though the motion equations do not include any stochastic noise terms. Fourier spectrum also has multiple peaks, including primary frequency *f*_t_ = 4.2 Hz, super-harmonics 2*f*_t_ = 8.4 Hz, subharmonics *f*_t_/3 = 1.4 Hz, and ultra-subharmonics 2*f*_p_/3 = 2.8 Hz and 4*f*_p_/3 = 5.6 Hz. Figure 6c shows the complex trajectory of chaotic motion in the phase plane. Figure 6d shows the Poincaré section of chaotic vibration. A stretching and folding structure, corresponding to chaos characteristics, is observed. These features serve as typical evidence for the existence of chaotic vibrations.

### Delayed feedback control

**Figure 7:**
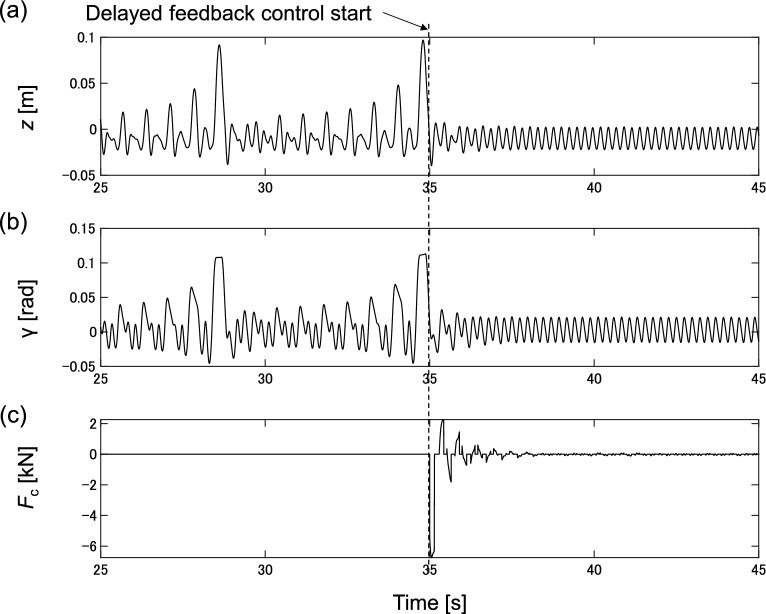
Delayed feedback control application results for forcing frequency *f*_t_ = 4.2 Hz; (**a**) Vertical displacement *z*; (**b**) Pitch angle *γ*; (**c**) Delayed feedback control force *F*_c_.

DF control is designed and applied to eliminate chaotic vibrations. Figure 7 shows the numerical results for the forcing frequency of *f*_t_ = 4.2 Hz with K_df_ = 55,000, and τ = 0.238 tuned by the trial-and-error. Figure 7a–c show the vertical motion *z*, pitch angle *γ*, and DF control force *F*_c_, respectively.

**Figure 8:**
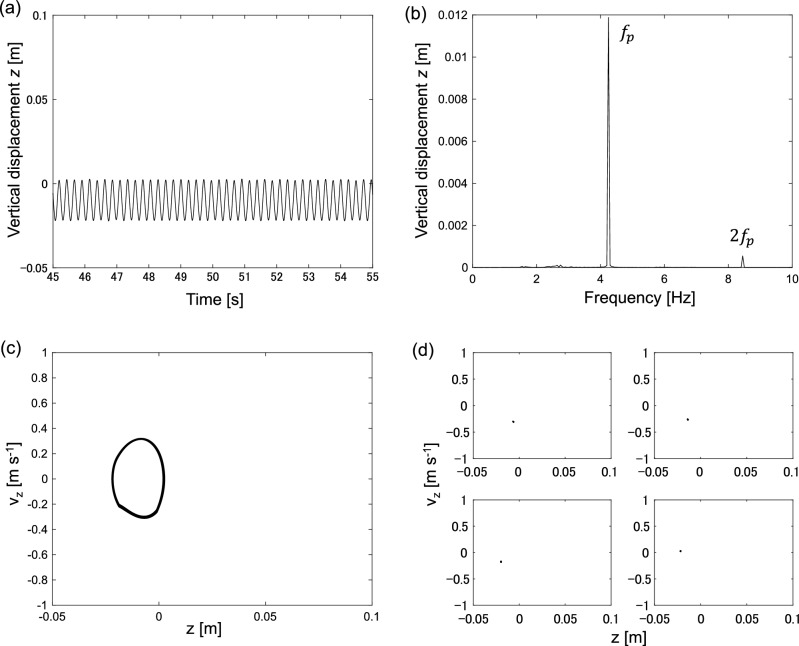
Delayed feedback control application results for forcing frequency *f*_t_ = 4.2 Hz; (**a**) Time series of vertical displacement, (**b**) Fourier spectrum of vertical displacement, (**c**) Phase plane spanned by vertical displacement *z* and vertical velocity *v*_z_, velocity, (**d**) Poincaré section spanned by vertical displacement *z* and vertical velocity *v*_z_.

The DF control force *F*_c_ is applied at 35.0 s. After applying the control force, the complex motion gradually stabilizes as it is converted into periodic motion. The DF control force *F*_c_ decreases as the motion becomes periodic. The vibration level is significantly decreased, and chaotic vibration is eliminated. Figure 8a–d show the time series of vertical displacement, Fourier spectrum of vertical displacement, phase space spanned by vertical displacement, velocity, and pitch angle, and Poincaré section, respectively.

Figure 8a shows that the vertical acceleration is stabilized as it is converted into periodic motion owing to DF control. Furthermore, the vibration level in Fig. 8a is smaller than that in Fig. 6a. A comparison of the Fourier spectra shown in Figs. 6b and 8b indicates that the DF control eliminates chaotic motion and reduces the noise level. Moreover, Figs. 8c and d indicate periodic motion. The period of the stabilized vibration corresponds to the inverse of the forcing frequency, which is 1/4.2 = 0.238 s in this case.

**Figure 9:**
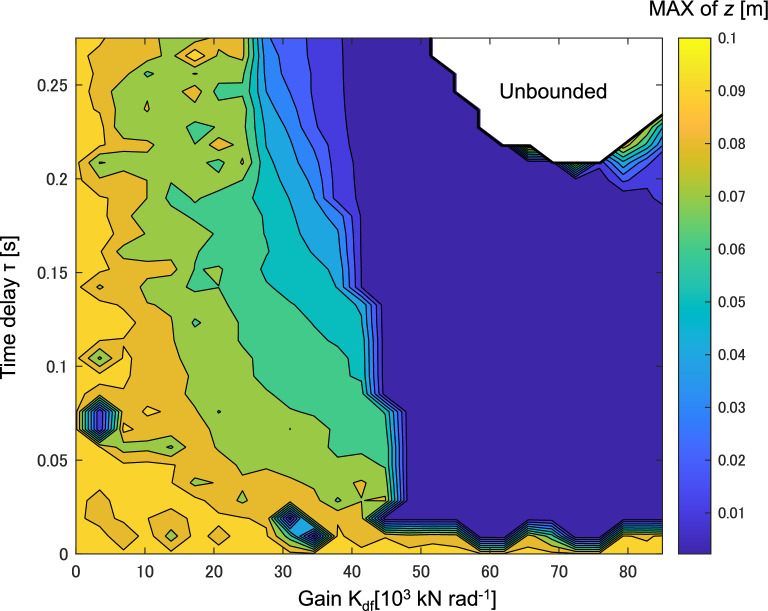
The maximum value of vertical displacement *z* at different control gain K_df_ and time delay τ.

To investigate the relationship between dynamic stability and the parameters of delayed feedback (DF) control, the values of K_df_ and τ are varied within the ranges of 0 to 85,000 and 0 to 0.28, respectively. Figure 9 illustrates the maximum vertical displacement value *z* obtained from each DF control simulation.

In Fig. 9, as gain K_df_ increases, the vibration level is reduced, and chaos is stabilized into periodic vibration. Regarding time delay τ, any time delay above 0.01 is able to suppress chaotic vibration. However, combination of larger K_df_ and τ can lead to unbounded or unstable results, as indicated by the white blank in Fig. 9. Therefore, for practical application, starting with smaller values of K_df_ and τ as the initial control tuning point and gradually increasing them to larger values can effectively suppress chaotic vibration. The period of the stabilized vibration is not affected by the DF control parameters. All stabilized periodic motion has a period equal to the inverse of the forcing frequency, which is 0.238 s for a forcing frequency of 4.2 Hz.

**Figure 10:**
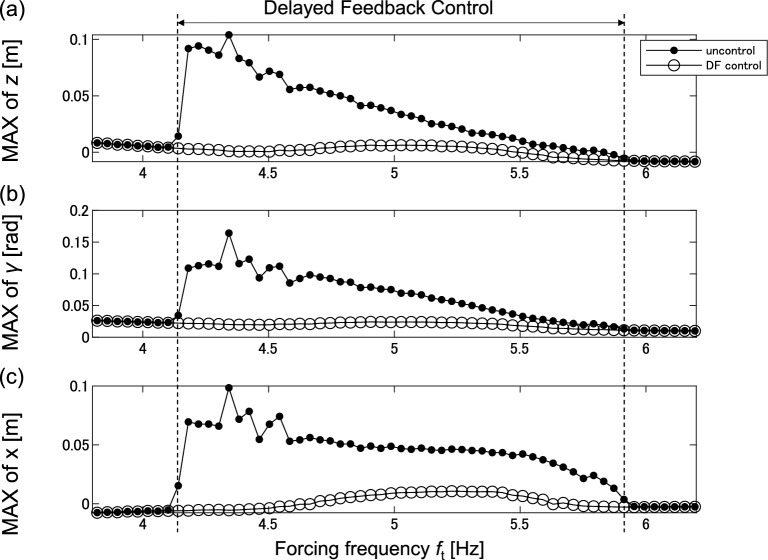
Comparison between uncontrol and DF control frequency response; the solid and hollow lines denote the uncontrol and control responses, respectively; (**a**) Frequency response of vertical displacement *z*; (**b**) Frequency response of pitch angle γ; (**c**) Frequency response of longitudinal displacement *x*.

To show the DF control effect in other frequency points, frequency response is conducted with DF control with K_df_ = 55,000, and τ = 0.238. Figure 10 compares the uncontrol and control frequency responses to investigate the vibration reduction effect of DF control. Figure 10a–c show the frequency responses of the vertical displacement *z*, pitch angle γ, and longitudinal displacement *x*, respectively.

DF control is applied in the range of 4.1–6.0 Hz to eliminate chaotic vibrations. The vibration level of the controlled frequency response curve is smaller than that of the uncontrolled frequency response curve. The results indicate that DF control is effective in reducing vibration levels and eliminating chaotic vibration.

## Conclusion

This study investigated the theoretical feasibility of DF control in eliminating chaotic vibrations in nonlinear impact dynamics of agricultural tractors. The DF control force was input as a longitudinal driving force on the rear wheel; it was designed based on the trial-and-error method. The frequency response, bifurcation diagram, and largest Lyapunov exponent λ_1_ were obtained to identify the parametric region in which chaotic vibration occurred. Delayed feedback control was applied to the agricultural tractor dynamics during the numerical simulation. The delayed feedback successfully eliminated chaotic vibrations and reduced the vibration level in the frequency response curve. The numerical results demonstrated the theoretical feasibility of delayed feedback control for agricultural tractors. This feasibility study is expected to contribute to improving the safety aspects of agricultural tractor operators and preventing damage to the tractors by reducing the risk of overturning.

Although the feasibility of applying DF control to agricultural tractors is clarified numerically in this research, there are several topics that should be explored in future research. In order to apply DF control to real agricultural tractors, it is important to consider factors such as road randomness and driver behavior models within the tractor model. In addition, it is necessary to validate the proposed DF control through field experiments. For a deeper understanding of the complex dynamics of agricultural tractors with DF control, simpler modeling approaches should be explored and advanced dynamic analyses should be conducted, including multistability and basin analysis. Considering the influence of random road surfaces, the equations of motion for the tractor become nonlinear differential equations with stochastic components, requiring intensive theoretical analysis for control design. Furthermore, conducting a comparison of different nonlinear control methods would be beneficial for selecting the most suitable control method for agricultural tractor dynamics.

## Data Availability

The datasets that support this study are available with the corresponding author and can be accessed upon reasonable request.
